# Decontamination of Petroleum-Contaminated Soils Using The Electrochemical Technique: Remediation Degree and Energy Consumption

**DOI:** 10.1038/s41598-018-21606-4

**Published:** 2018-02-19

**Authors:** Constantin Streche, Diana Mariana Cocârţă, Irina-Aura Istrate, Adrian Alexandru Badea

**Affiliations:** 1 Bucharest, Faculty of Power Engineering, University POLITEHNICA of Bucharest, Splaiul Independentei 313, sector 6, Bucharest, 060042 Romania; 2 Bucharest, Faculty of Biotechnical Systems Engineering, University POLITEHNICA of Bucharest, Splaiul Independentei 313, sector 6, 060042 Bucharest, Romania; 3 Academy of Romanian Scientists, Splaiul Independentei 54, RO-030167 Bucharest, Romania

## Abstract

Currently, there are different remediation technologies for contaminated soils, but the selection of the best technology must be not only the treatment efficiency but also the energy consumption (costs) during its application. This paper is focused on assessing energy consumption related to the electrochemical treatment of polluted soil with petroleum hydrocarbons. In the framework of a research project, two types of experiments were conducted using soil that was artificially contaminated with diesel fuel at the same level of contamination. The experimental conditions considered for each experiment were: different amounts of contaminated soils (6 kg and 18 kg, respectively), the same current intensity level (0.25A and 0.5A), three different contamination degrees (1%, 2.5% and 5%) and the same time for application of the electrochemical treatment. The remediation degree concerning the removal of petroleum hydrocarbons from soil increased over time by approximately 20% over 7 days. With regard to energy consumption, the results revealed that with an increase in the quantity of treated soil of approximately three times, the specific energy consumption decreased from 2.94 kWh/kg treated soil to 1.64 kWh/kg treated soil.

## Introduction

Anthropogenic activities can often negatively affect the water, air and soil quality with real risks to human health^1–3^. The need to restore the geological environment by cleaning, remediation and/or ecological reconstruction of contaminated soils calls for the restoration of the natural quality of the affected geological environment, restitution of its functions, and elimination or reduction of actual or potential risks to human health and the environment^4–6^.

Spilling substances, such as petroleum hydrocarbons, on the ground changes the chemical properties of the soil with regard to the amount of organic carbon, which increases (75% of the carbon in oil is oxidable); the pH decreases because of the increase in the quantity of organic carbon and organic acids, while the interchangeable Fe and Mg contents increase, as does the availability of phosphorus^7^.

On the other hand, petroleum hydrocarbons that are in contact with the soil form an impermeable coating at the surface, which prevents water circulation in soil and gas exchange between the soil and air, causing the roots of plants to suffocate. As the soil becomes anaerobic, the metabolic activity and number of bacteria decrease^8^. Various types of hydrocarbons are associated with soil organic matter, and therefore, their absorption varies according to the nature and content of organic substances in the soil and chemical processes that may occur. The result is an alteration of the composition of hydrocarbons spilled onto the soil. The soil composition and structure and its humidity, nature of organic matter, and structure and quantity of the contaminating oil product give the soil-pollutant system different physicochemical features. Clearly, the hydrocarbons that are most absorbed and adsorbed by the organic matter in the soil are the most resistant to losses or alterations by other processes. By contrast, volatile hydrocarbons (soluble) are susceptible to changes due to volatilization, filtration and biodegradation^9^.

In Europe, the issue of soils contaminated with petroleum products and their remediation are among the most complex tasks in the environmental protection field in terms of financial and organizational aspects.

Even if there are different remediation methods for contaminated sites, they fail to deliver consistent results when the contaminated area is located at a considerable depth and the soil type is clay due to its low permeability. Moreover, pollutants, such as petroleum hydrocarbons, have a high adsorption rate compared to soil particles, making their removal or destruction more difficult^10–12^.

The electrochemical remediation method has been called an electrokinetic treatment, 1electroremediation treatment, and electrochemical remediation treatment over time. The method is classified as a continuous current technology, known as Direct Current Technologies - DCT. These technologies have been initially used to remove metals, radionuclides and polar inorganic pollutants from soil and groundwater. The principle of electrochemical remediation consists of applying an electrical potential difference to electrodes, or to a network of electrodes, inserted in different configurations in the contaminated soil. Usually, the applied electric potential is over the range of a few volts per centimeter (1 V/cm), while the current density is over the range of milliamperes per square centimeter (1 mA/cm^2^)^13–15^. When the current flows through the soil, it causes different physical and chemical phenomena that underline the technologies of continuous current (DCT), namely, electrolysis, electroosmosis, and electrophoresis, changes in the pH and water hydrolysis^16^. DCT technologies include two types of processes, namely, electrokinetic transport (electro-osmosis, electromigration and electrophoresis – phenomena that help the transport, mobilization and concentration of pollutants) and electro-oxidation, which is based on redox reactions that are electrochemically induced (responsible for the mineralization of immobile organic contaminants)^13,17^.

The toxicity of different types of pollutants may be substantially reduced by continuous current technology due to oxidation and reduction processes^18^. Consequently, the electrochemical method targets contaminants, such as metals^19^, anions and organic matter, in the soil, mud and sludge. Various surveys have highlighted the applicability of this method with good results for the decontamination of soils or sediments polluted with different toxic contaminants, such as polycyclic aromatic hydrocarbons (PAHs), chlorinated solvents^13,16,20,21^ and chlorophenols^22^. Different studies have shown that application of electroremediation can mineralize a variety of organic compounds with low power consumption^13^. Additionally, other research studies have shown that electrochemical oxidation is an alternative method for transferring benzene to p-benzoquinone, which is an important chemical that can be found in various dyes, insecticides and fungicide, in the polymer industry and as a toner and intensifier in photographic industry^23^. The applicability of direct current technologies for the remediation of contaminated sites is effective due to the physical and chemical processes that occur when the electric current flows through soil subject to remediation. In recent years, more researchers have been studying DCT and their efficiency in removing organic pollutants from soils and sediments. These studies suggest that DCT can be effectively used to mineralize several organic substances with lower power costs^16,24^. Xiaolin Li *et al*. consider electrochemical oxidation to be an environmentally friendly and promising method that can offer some advantages in terms of compatibility, energy efficiency, low volume application, versatility and amenability to automation^23^.

According to Yeung and Ying-Ying Gu^25^, oil-contaminant interactions are soil specific, contaminant-specific, dynamic, reversible, and pH dependent. The authors note that if electrochemical reactions and soil-contaminant interactions are considered at the same time, the electrochemical remediation process becomes extremely complex^25^, which is why supplementary research on electrochemical reactions and the soil-contaminant interactions that take place, together with an evaluation of different important parameters that influence the success of electrochemical remediation (such as soil pH), is essential to achieve good results in the removal of organic compounds using the electrokinetic method. Consequently, in this study, an anode-cathode separated electrochemical remediation system was employed. The efficiency of the treatment was evaluated based on the amount of organic pollutants removed and the impact of the technology on soil properties (pH, conductivity, moisture content, total dissolved solids -TDS). At the same time, the energy consumption was measured. Another objective of the research was to analyze the influence of the number of power sources on the energy consumption.

## Material and Methods

### Soil samples and experimental setup

**Figure 1:**
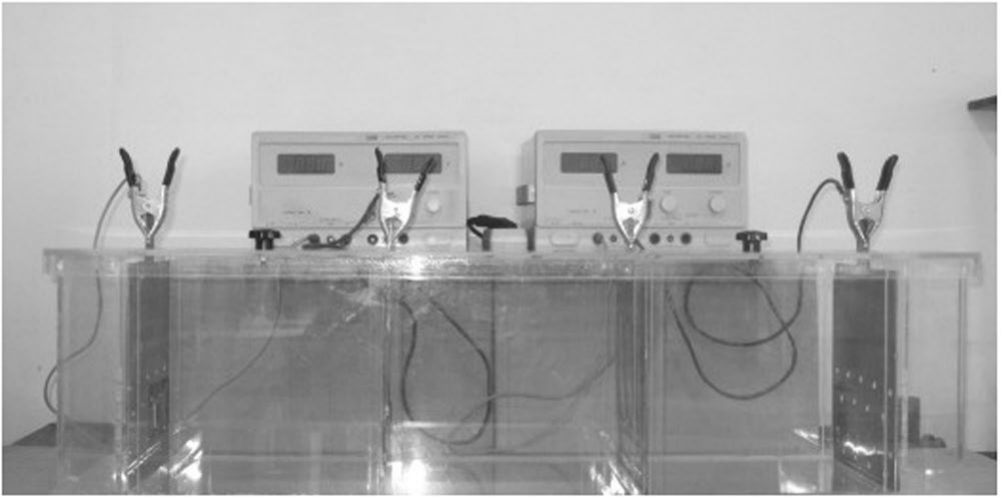
General view of the experimental setup.

**Figure 2:**
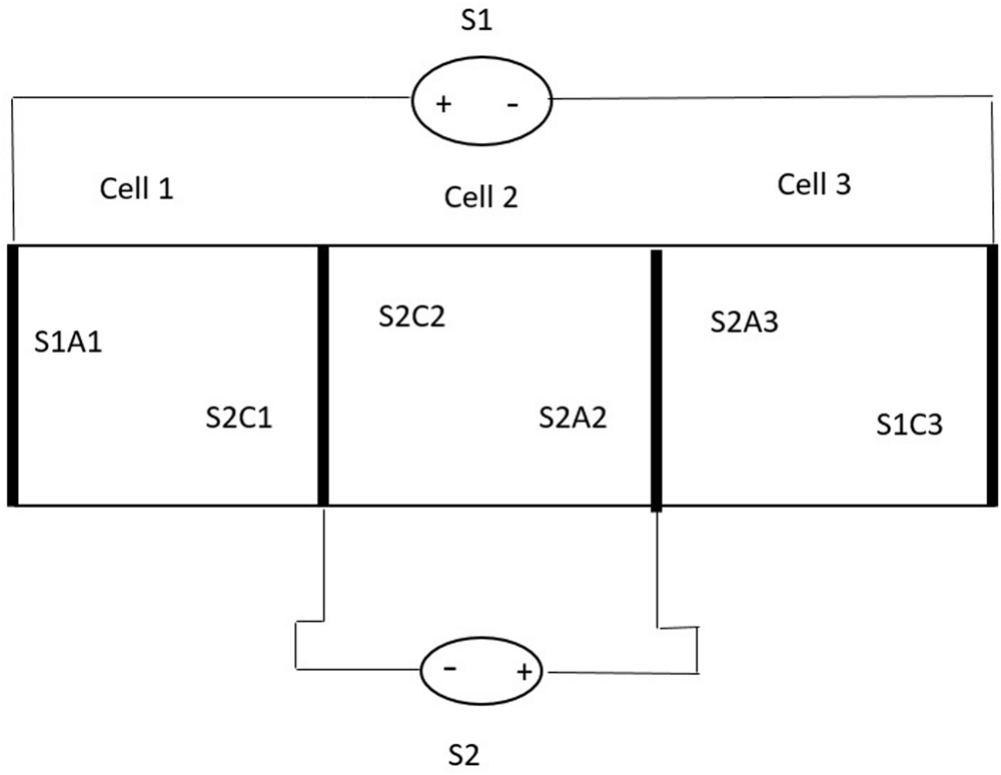
Schematic representation of the experimental setup (S1, S2 – continuous current source 1; S1A1 – anode of source 1 in cell 1; S2C1 – cathode of source 2 in cell 1; electrodes indices 1, 2, 3 refer to the cell to which they belong).

To test the electroremediation method, an experimental setup was used (Fig. 1) that included the following components: an electrochemical cell, power supply and electrodes (an anode and a cathode one). The schematic representation of the experimental setup is illustrated in Fig. 2.

Artificial contamination of soil with diesel fuel was performed in a controlled environment. The artificial contamination was carried out by adding diesel fuel to the soil sample at a quantity needed to reach the pre-established initial concentrations. Afterwards, the contaminated soil was left for 3 days to assure good homogenization and was then placed in the experimental setup (Fig. 1).

The initial soil moisture content was only 3.61%. Since it is commonly known that soil moisture has an important role in facilitating the flow of an electrical current through it, an additional quantity of water was introduced to obtain a moisture content of over 20%. This ensured a good environment that stimulated electrochemical processes. Plate electrodes were installed at the ends of the experimental installation, and the soil placed in the electrochemical cell between the two electrodes was compacted to reach a density of 1.5 g/cm^3^. Electrodes made of stainless steel with an area of 200 cm^2^ were used. This setup avoided the formation of air pockets in the soil and led to a density that was close to the normal average density of soils in real situations.

The properties of the soil used in the experiment, identified as Luvisols, are presented in Table 1. Table 2 illustrates the results from the granulometric analysis.

**Table 1:** Properties of diesel-contaminated soil used for the experimental tests.

Soil properties	PH	Nt	Humus	P_AL_	K_AL_	The mobile forms of microelements				The total soluble salts content
						Zn	Cu	Fe	Mn	
Units of measurement	—	%		mg kg^−1^						mg/100 g sol
Identified values	6.97	0.149	3.18	110	364	6.54	4.78	12.9	51.1	16

**Table 2:** Grain size analysis.

Particle size (mm)	Percentage (%)
4	0.615
2	6.365
0.8	25.589
<0.8	67.431

### The experimental framework

The experiments involved treating two different quantities of soil, 6 kg and 18 kg of contaminated soil. The electrochemical treatment was carried out over a period of 14 days. Three concentration levels of the pollutant (diesel fuel) were tested: 10 g diesel fuel/kg soil, 25 g diesel fuel/kg soil and 50 g diesel fuel/kg soil. For each concentration, two types of tests were carried out, each with a different constant value for the electric intensity: 0.25 A and 0.5 A. Therefore, in total, there were 12 tests analyzed (6 tests for each soil amount).

The working phases developed for each single experiment were: soil insertion in the electrochemical cell; establishing connections between the electrodes (that were already in the electrochemical cell) and power supply; setting up the main operating parameters, such as a constant electric intensity; sampling to determine the initial soil characteristics; and powering up the power supply.

If, for the first tests (considering 6 kg of contaminated soil), only cell 2 was used (see Fig. 2), the following dimensions were used: 250 × 200 × 100 mm (L × l × h). For the tests performed with 18 kg of polluted soil, the dimension were 750 × 200 × 100 mm (L × l × h), which were achieved by connecting two power supplies. For each test, the initial pollution was approximately 1%, 2.5% and 5%.

All of the experiments had a treatment period of 14 days. Monitoring was performed at the beginning, middle (after 7 days) and end of the performed tests.

### Analytical methods

Across the experiments, the following parameters were followed: soil pH, redox potential (ORP), electroconductivity (EC), resistivity (ρ), TDS, soil moisture, intensity of the electric current (I), voltage of the electric current (U) and concentration of total petroleum hydrocarbons (TPH).

pH, redox and temperature were monitored in a soil/water suspension using IQ SENSOR NET pH/ORP sensors connected to a multiparameter meter, 2020XT. Therefore, an important monitored parameter in the framework of the research was the ORP (oxidation reduction potential or redox). This parameter provided information about the area with the highest level of oxidation. Because the objective was to stimulate oxidation reactions over a larger area, during the test, the potential was changed when the ORP value was near or below 0 mV. A negative value of ORP is an indication that oxidation reactions, which are responsible for the mineralization of certain organic compounds, no longer occur. The potential change involved a change of the roles of the electrodes (the anode became the cathode, and vice versa). By changing the electrical load of the two electrodes, the electrically induced chemical processes were reversed, and the electroremediation process was resumed^26–29^.

The method for determining the moisture content of soil was oven drying. A weighted quantity of soil was placed in an oven at 105 °C for 24 h. Then, dried soil was weighed again, the moisture content of soil to be determined.

The current and voltage applied were monitored with multiparameter equipment (model UNI-T) by connecting the sensors to the electrodes or the wires that were connected to the power supply.

The EC, ρ and TDS parameters were monitored with a C863 Multi-Parameter Analyzer Dexktop Meter – C863 by using approximately 50 g of contaminated soil introduced on glass and mixed with 200 ml of demineralized water. The ratio of this solution, which was used also for the pH measurement, was 1:5 (soil: demineralized water).

To determine the TPHs in soil samples, chemical analysis was performed according to SR 13511/2007 (ASRO 2007) (the Romanian standard)^30^. For the extraction of diesel fuel, dichloromethane (CH_2_Cl_2_) was used as a solvent. For this determination, a Classic SOXHLET Apparatus and rotary evaporator, model P/N Hei-VAP Value/G3: 560-01300-00, were used (both Heidolph producer).

## Results and Discussion

In this study, two types of tests were performed: IDER 1, for tests performed in 6 kg of contaminated soil, and IDER 2, for tests using 18 kg of contaminated soil. For each type of experiment, two levels of constant electric intensity (0.5 A and 0.25 A) and three levels of pollutant concentrations (1%, 2.5% and 5%) were evaluated. The main characteristics of the tests are indicated with capitals letters in Table 3.

**Table 3:** The main parameters of the tests.

Experiment	Initial pollutant concentration	Current intensity [A]
A	1%	0.5
B	1%	0.25
C	2.5%	0.5
D	2.5%	0.25
E	5%	0.5
F	5%	0.25

Because the initial moisture for the contaminated soil samples was quite low, a certain amount of water was added to increase the moisture to approximately 20%. To maintain a constant level of electric intensity, the voltage ranged between 10 V and up to 130 V for IDER 1. According to Ohm’s law, when the resistance in the medium is the same, the voltage increases with an increasing current. The same trend was observed across the current experiments: the resistance in the soil started to increase due to the depletion of ions from the soil^31^.

**Figure 3:**
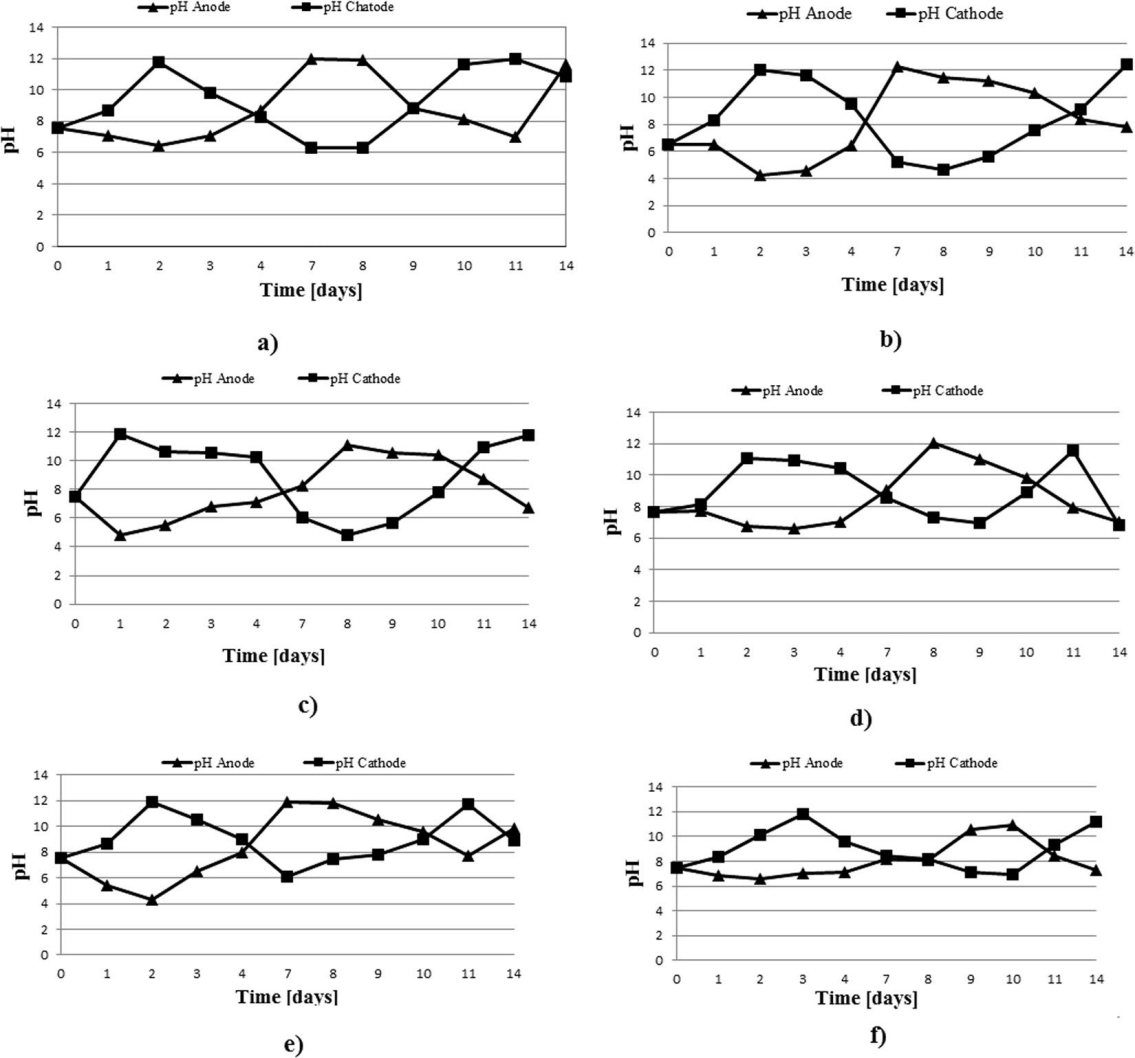
pH trend during all of the IDER 1 tests: (**a**) IDER 1-A; (**b**) IDER 1-B; (**c**) IDER 1-C; (**d**) IDER 1-D; (**e**) IDER 1-E; (**f**) IDER 1-F.

**Figure 4:**
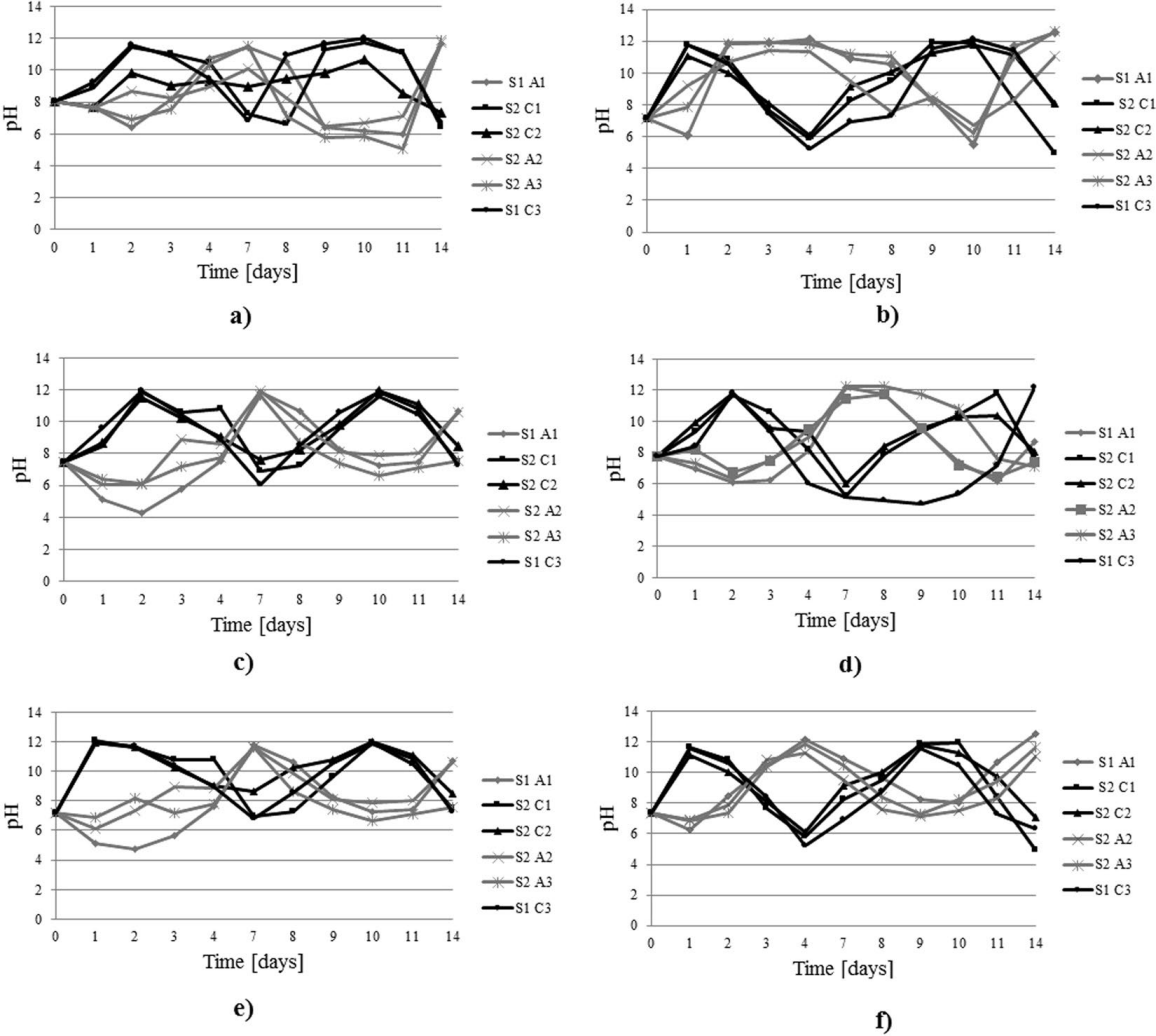
pH trend during all of the IDER 2 tests: (**a**) IDER 2-A; (**b**) IDER 2-B; (**c**) IDER 2-C; (**d**) IDER 2-D; (**e**) IDER 2-E; (**f**) IDER 2-F.

The first monitored parameter was the soil pH in the anode and cathode areas. The pH variation in the two areas of interest followed the trend specified in the literature^16,32^, i.e., acidification in the anode area and basification in the cathode area. The soil pH was monitored throughout the test and showed the occurrence of electrolysis reactions (Fig. 3 for IDER 1 and Fig. 4 for IDER 2). Consequently, the soil in the anode area became increasingly acidic, and the soil in the cathode area became increasingly basic.

**Figure 5:**
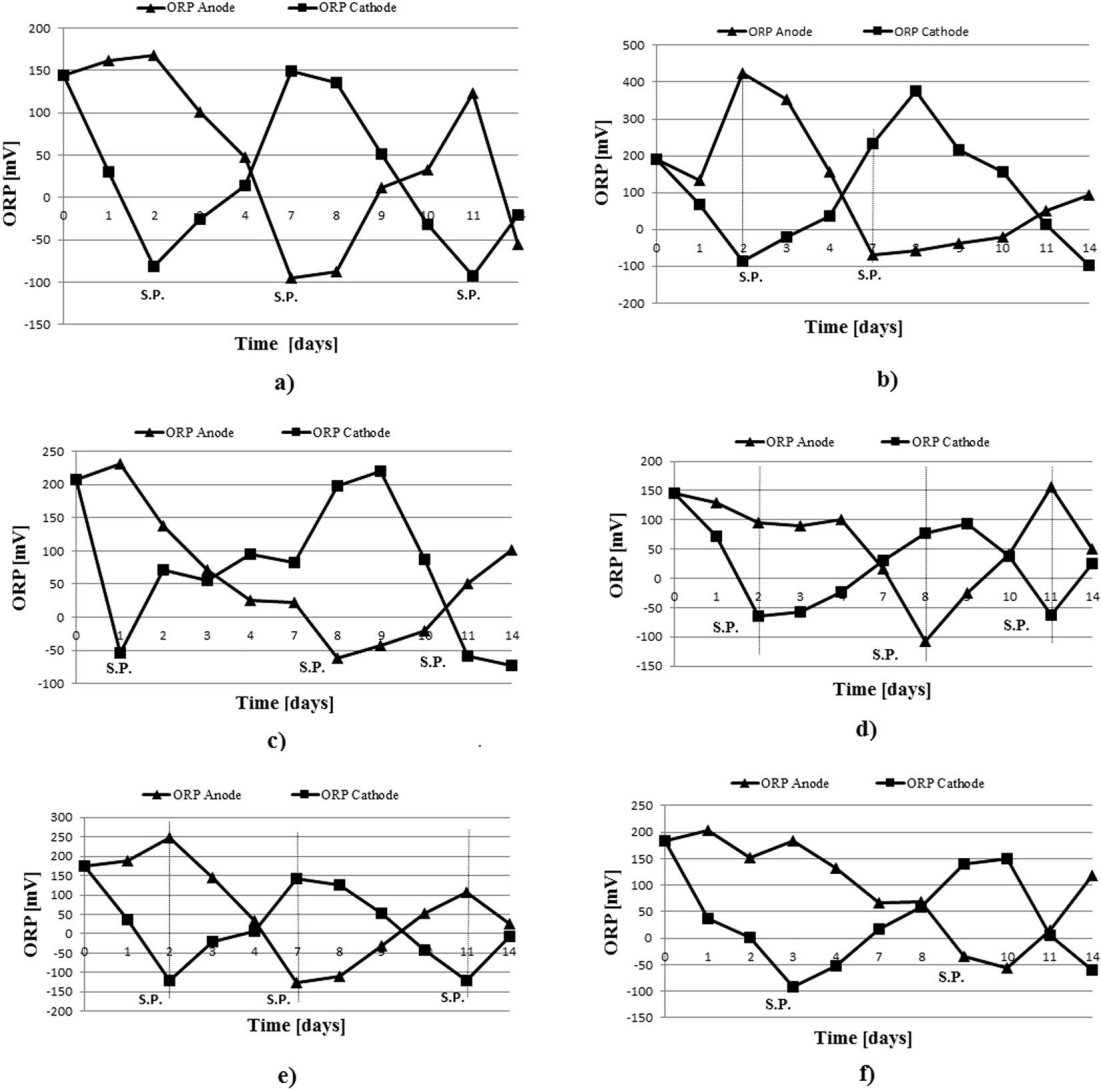
ORP trend during all of the IDER 1 tests1: (**a**) IDER 1-A; (**b**) IDER 1-B; (**c**) IDER 1-C; (**d**) IDER 1-D; (**e**) IDER 1-E; (**f**) IDER 1-F.

**Figure 6:**
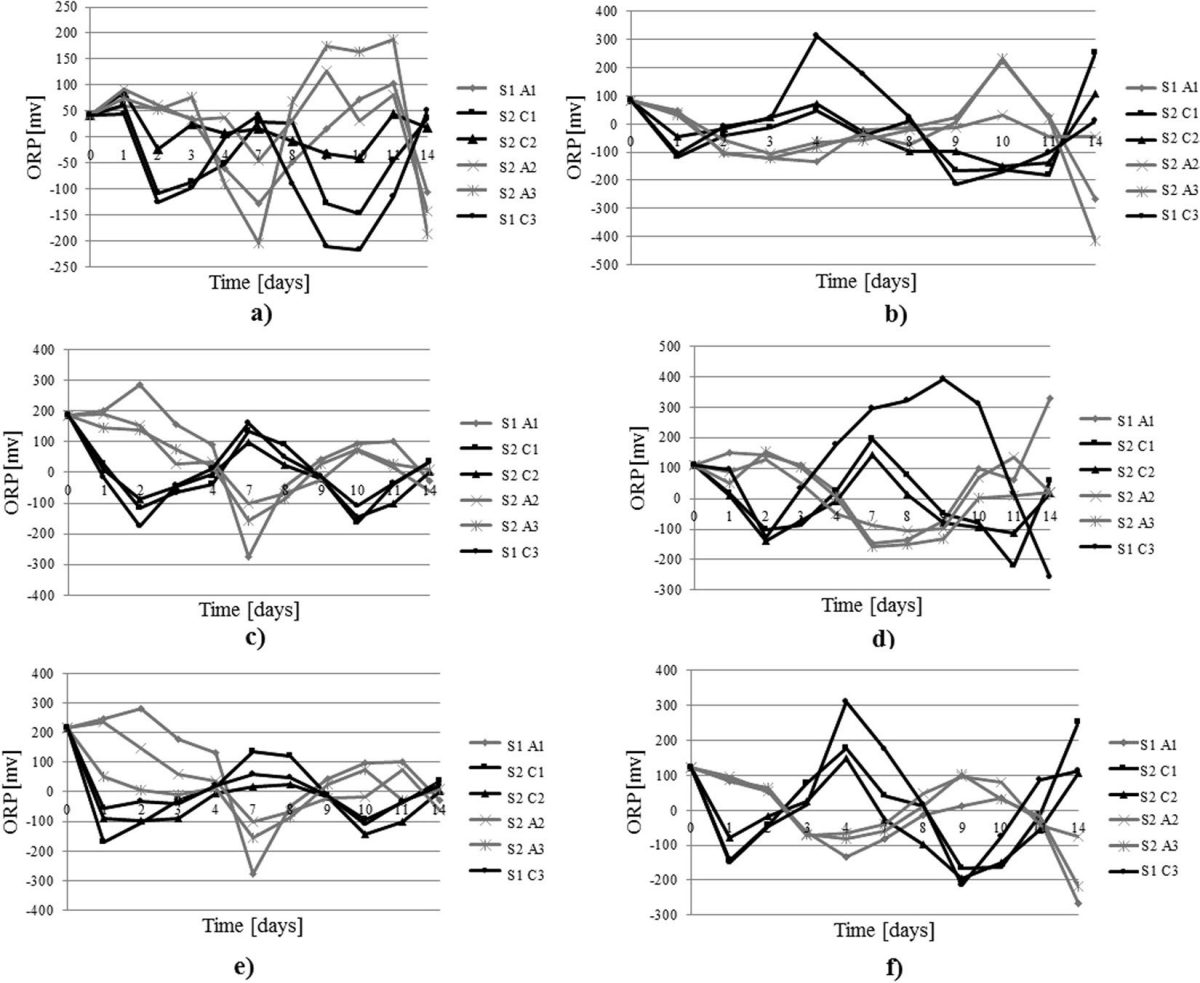
ORP trend during all of the IDER 2 tests: (**a**) IDER 2-A; (**b**) IDER 2-B; (**c**) IDER 2-C; (**d**) IDER 2-D; (**e**) IDER 2-E; (**f**) IDER 2-F.

Regarding the ORP, according to Ren *et al*., this parameter is considered to be one of the most important parameters. As observed by the cited author, oxidation-reduction reactions are the most well-known solution to treat and degrade organic contaminants in soil environments^31^. The ORP trend during the application of the electrochemical treatment is illustrated in Figs 5 and 6. In these figures, the polarity shifts are dotted with vertical bars and denoted by the letters S.P. (polarity change). Following analysis of the obtained results, it was observed that the specific reactions of the electrokinetic processes occurred immediately after the start of the test, as evidenced by the important change in the ORP initial values. When the redox potential has a value near 0 mV or below 0 mV, a potential change is needed to stimulate the oxidation reactions that usually take place near the anode area. In this way, oxidation reactions are induced in the entire soil sample. During the test, a polarity change was needed twice, leading to an alternation tendency in the two areas where the electrical charge was applied. Moreover, the redox potential is the parameter that guides the electroremediation treatment with a polarity change^33^.

**Figure 7:**
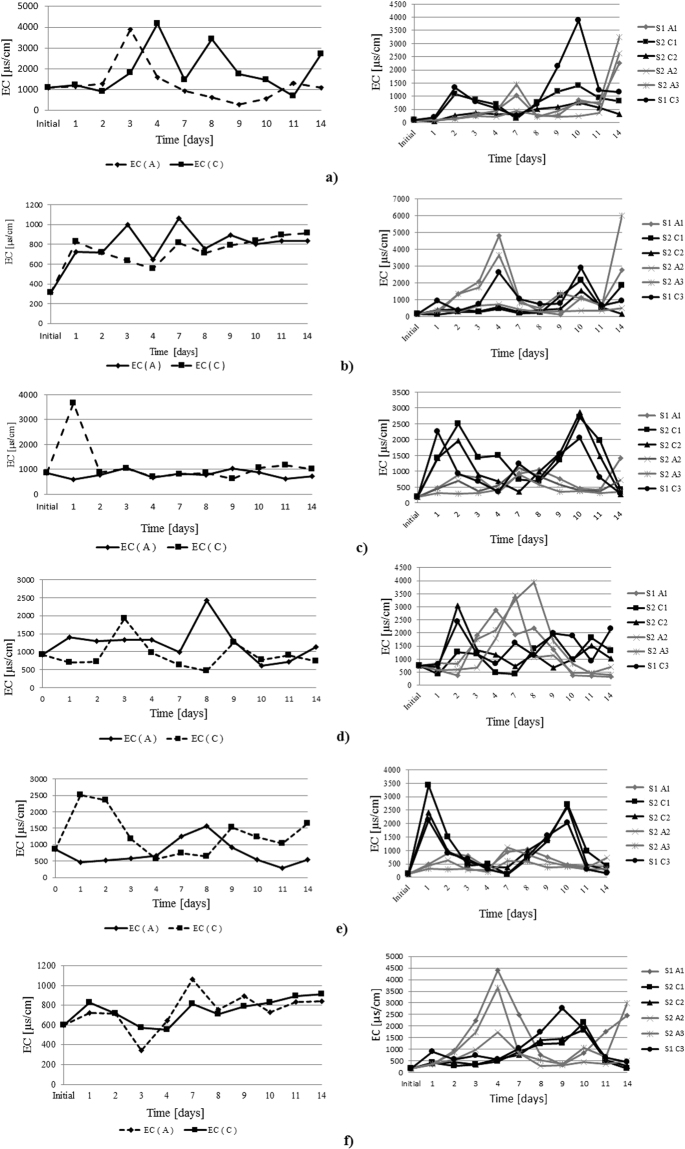
Electroconductivity trend during the experimental research; IDER 1 experiments are presented in the left column and IDER 2 experiments are presented in the right column: (**a**) 1% pollutant concentration and 0.5A; (**b**) 1% pollutant concentration and 0.25A; (**c**) 2.5% pollutant concentration and 0.5A; (**d**) 2.5% pollutant concentration and 0.25A; (**e**) 5% pollutant concentration and 0.5A; (**f**) 1% pollutant concentration and 0.25A.

**Figure 8:**
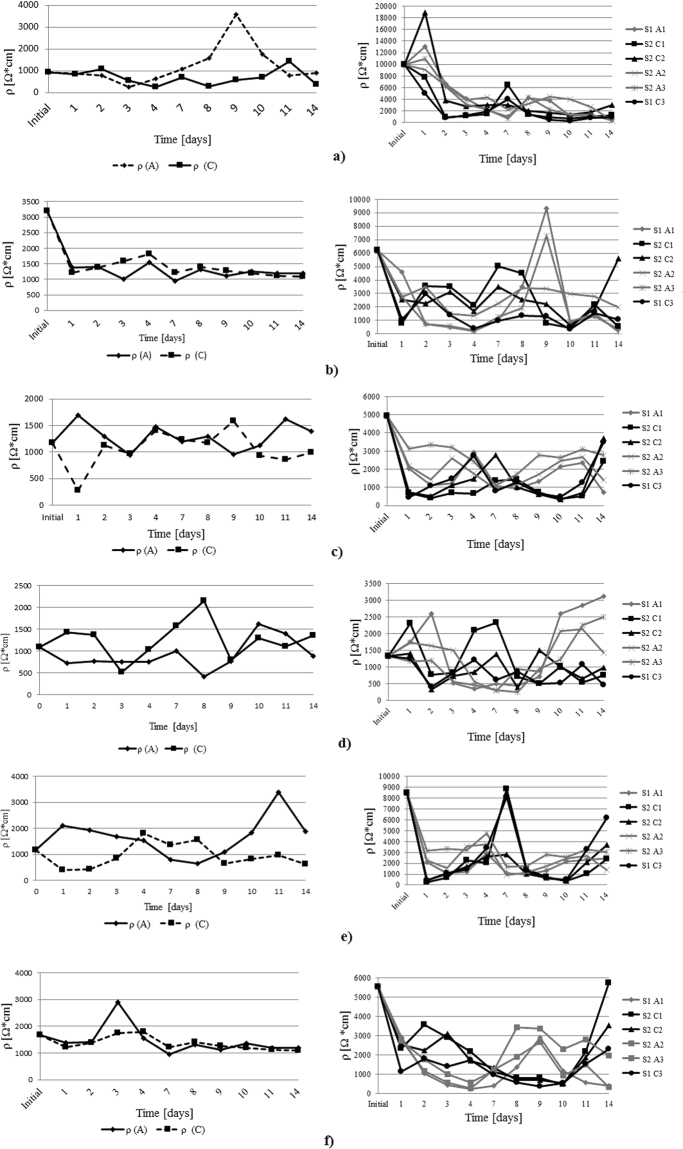
Resistivity trend during the experimental research; IDER 1 experiments are presented in the left column and IDER 2 experiments are presented in the right column: (**a**) 1% pollutant concentration and 0.5A; (**b**) 1% pollutant concentration and 0.25A; (**c**) 2.5% pollutant concentration and 0.5A; (**d**) 2.5% pollutant concentration and 0.25A; (**e**) 5% pollutant concentration and 0.5A; (**f**) 1% pollutant concentration and 0.25A.

**Figure 9:**
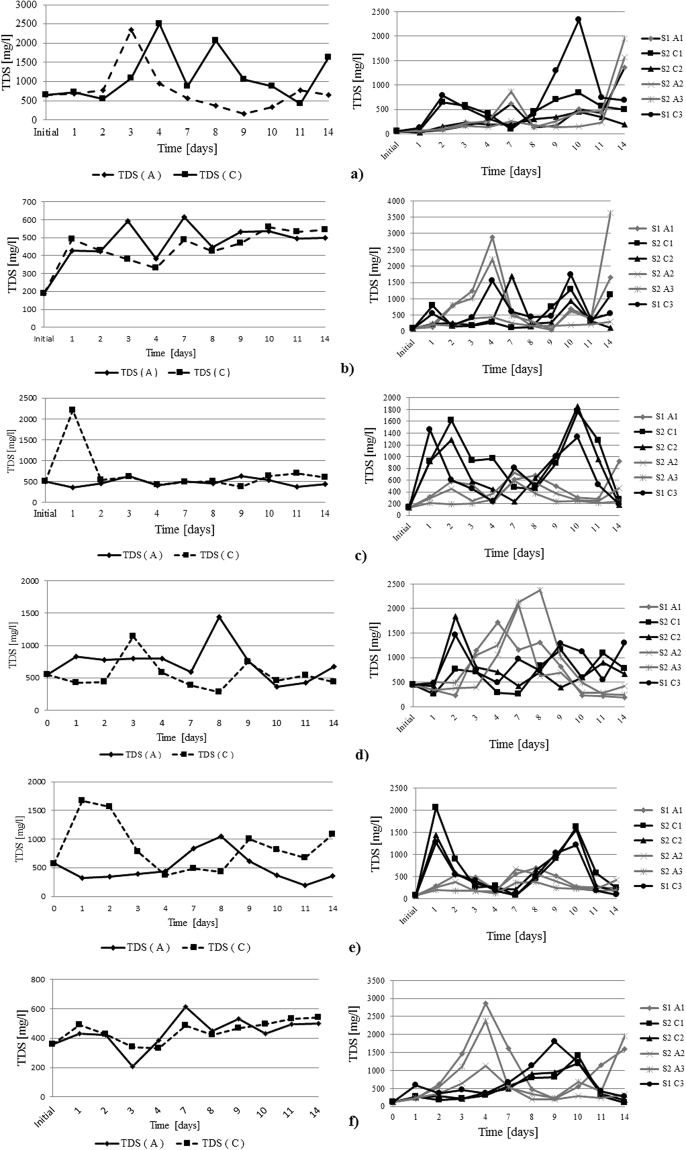
Total dissolved solids trend during the experimental research; IDER 1 experiments are presented in the left column and IDER 2 experiments are presented in the right column: (**a**) 1% pollutant concentration and 0.5A; (**b**) 1% pollutant concentration and 0.25A; (**c**) 2.5% pollutant concentration and 0.5A; (**d**) 2.5% pollutant concentration and 0.25A; (**e**) 5% pollutant concentration and 0.5A; (**f**) 1% pollutant concentration and 0.25A.

During the experiments, EC, ρ and TDS were also monitored. The results are presented as a comparison between the two types of experiments (Figs 7, 8 and 9).

Even if the electrokinetic removal of pollutants from soils is a promising *in-situ* remediation technology^34^, the success of this technique is highly dependent on the ability of the pollutants to migrate under the action of the electric field^35^.

Therefore, along with the pH, the electrical conductivity plays an important role in electrochemical processes. As already known, when the electrical conductivity is low, the current decreases and higher applied potentials are needed to avoid the passivation of the electrode, thus increasing energy consumption^36,37^.

Regarding resistivity, from Fig. 8, it is observed that for a smaller quantity of contaminated soil (IDER 1 tests), the values of this parameter are 4 to 6 times lower than for the IDER 2 tests. For the IDER 2 test with the experimental conditions of a 1% pollutant concentration and 0.5 A, the maximum value of the resistivity encountered during the experimental research was approximately 19000 Ω*cm.

Throughout the experiments, the TDS evolution was quite interesting: first, the TDS increased in the anode area, after which, due to the change in polarity, at the end of the experiment, the values of TDS were more or less the same for the two types of electrodes. For the tests in which 0.25 A was applied, the values for TDS were almost half those with a double current intensity.

**Figure 10:**
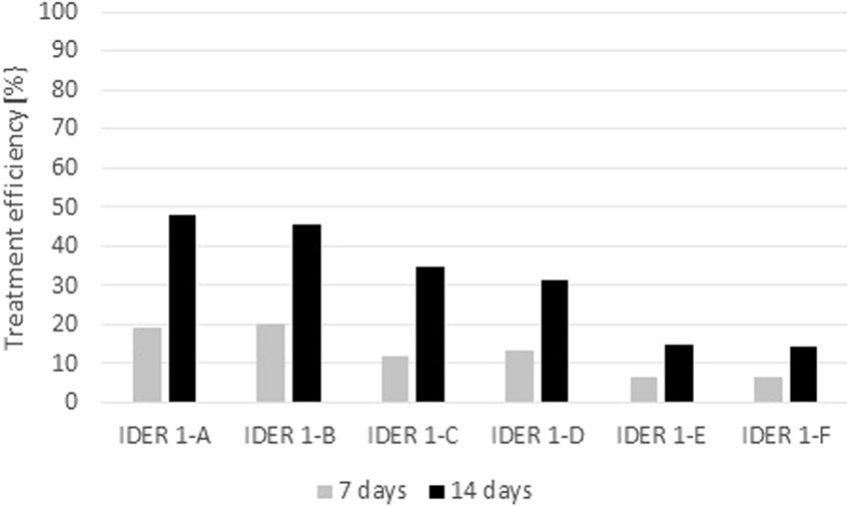
Treatment efficiency for the IDER 1 tests.

**Figure 11:**
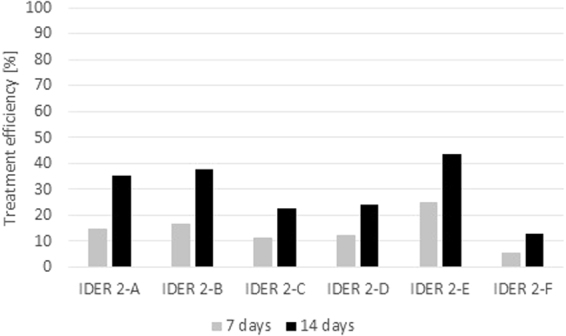
Treatment efficiency for the IDER 2 tests.

An important indicator that is generally used to assess the degree of remediation for a certain applied remediation method is to determine the concentration of the pollutant before and after application of the remediation treatment. As specified above, in the framework of the experiments, two areas were monitored, namely, the anode and the cathode areas, for each cell. The results obtained after 7 days of monitoring, as well as after 14 total days of the applied treatment, are presented in Fig. 10 for IDER 1 and Fig. 11 for IDER 2. The average values of the contaminant concentrations in the soil for each test were considered.

Taking into account that the treated quantity of contaminated soil from IDER 2 was three times higher than that from IDER 1 (Figs 7 and 8), there were some differences regarding the obtained results. Since the tendency is that the efficiency increases over time, the differences can be attenuated by maintaining the treatment for a longer period of time (in case that an important soil quantity needs remediation) or through the use of an electrochemical treatment in combination with chemical oxidation. This is an approach that has become increasingly viable, as shown also by other authors. For instance, Yoo *et al*.^38^ obtained the following results for the removal of TPH after a series of batch test using different oxidants: with ozone, a 53.2% remediation degree; with hydrogen peroxide (H_2_O_2_), a 72.9% remediation degree; and with potassium permanganate (KMnO_4_), a 67.5% remediation degree.

Energy consumption is another extremely important parameter for evaluating a remediation technology. Therefore, during the experiments, consumption was measured by an electricity meter – type KGS02-01/1109. According to Sires *et al*.^37^ and Narong *et al*.^39^, energy consumption can also be determined according to equation (1):

1 $$P=\int V\cdot I\,dt$$

where P (kWh) is the energy consumption, V (V) is the voltage, I (A) is the current and t (hr) is the time. To determine the specific energy consumption, the above equation was divided by the total amount of the decontaminated soil.

With regard to the energy consumption, it was shown that an increase of the treated soil quantity of approximately three times was coupled with a decrease in the specific energy consumption from 2.94 kWh/kg treated soil to 1.64 kWh/kg treated soil. On the other hand, the energy consumption can be sustained by green energy, such as wind, tidal or solar energy, or by using biogas from an anaerobic digestion reactor to produce energy for the electrochemical reactor. Thus, incorporating renewable energy sources into the electrochemical treatment of organic polluted soils is a promising future prospect in this field of research.

The electrode type can also influence the energy consumption. According the literature^32^, despite the better removal rates obtained by Al electrodes, Fe electrodes are energetically more efficient than aluminum.

A key factor in the electrochemical process is the operating current density since it exerts a significant influence on the reaction kinetics and energy consumption. By increasing the current density, the extent of the anodic dissolution of consumable electrodes increases and results in an increase in hydroxide flocs, which promote the removal of pollutants^32,39^.

In summary, the use of a high applied current intensity greatly increases the energy consumption of the process without considerably improving the treatment efficiency^36^. Thus, the amount of current applied to the electrodes must be carefully determined to avoid additional energy consumption. Increasing the distance between the electrodes significantly raises the energy costs of the electrochemical process, and therefore, the minimum distance between the electrodes should be selected.

In Table 4, the main results obtained for tests performed for IDER 1 are illustrated.

**Table 4:** The main results achieved in the framework of the experimental research.

Test	Treatment efficiency (%)	Power Consumption (kWh)	Amount of diesel fuel removed (g)	Specific consumption (kWh/g of removed contaminant)
IDER 1-A	47.81	22.7	28.69	0.79
IDER 1-B	48.59	12.8	29.15	0.44
IDER 1-C	34.62	23.1	51.93	0.44
IDER 1-D	31.17	11.7	46.76	0.25
IDER 1-E	15.01	22.9	45.03	0.51
IDER 1-F	14.45	12.8	43.35	0.29
IDER 2-A	35.61	34.2	64.10	0.53
IDER 2-B	37.80	23.9	68.04	0.35
IDER 2-C	22.67	35.5	102.01	0.35
IDER 2-D	24.08	25.0	108.36	0.23
IDER 2-E	14.72	35.9	132.48	0.27
IDER 2-F	12.70	23.2	114.3	0.20

The average electricity consumption measured at the end of the IDER 1 experiments was approximately 17.67 kWh, which corresponds to an average specific consumption of 2.94 kWh/kg of remediated soil. For the IDER 2 experiments, the average energy consumption was approximately 29.6 kWh, which corresponds to an average specific energy consumption of approximately 1.64 kWh/kg of remediated soil.

**Figure 12:**
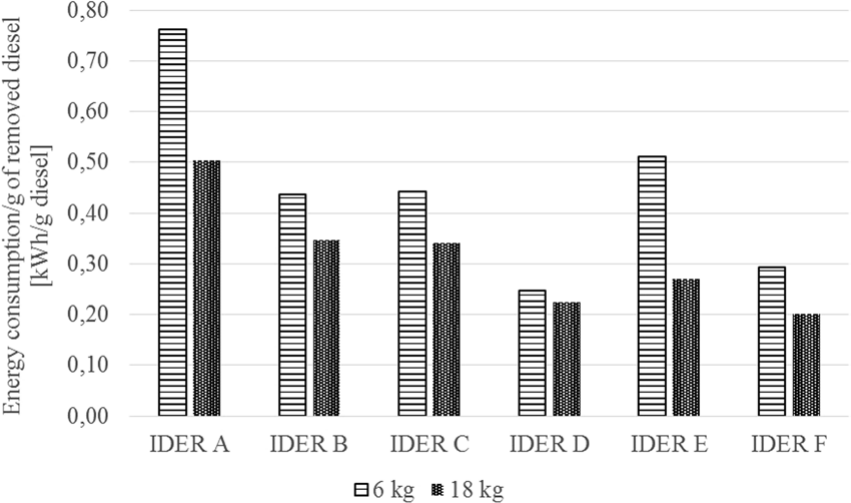
Comparison between the results obtained for the two sets of tests (IDER 1–6 kg and IDER 2–18 kg) regarding the specific energy consumption.

In Fig. 12, a comparison between the two experiments is shown, taking into account the specific energy consumption for each gram of removed contaminant.

Figure 12 shows that even though the treated soil quantity is three times larger, the specific energy consumption for each gram of removed pollutant has a better result in the IDER 2 tests (with respect to the treated soil quantity).

Because the distance between the two electrodes was only 250 mm and because the difference between the anode and cathode levels of concentration were not as high, we used the average value for the new concentration level to characterize the treatment efficiency.

## Conclusions

The issue of contaminated soils and the choice of the most appropriate remediation strategy is a current concern throughout the world. Important contaminants at the national and international levels are petroleum hydrocarbons. In this context, the main aim of the present research was to evaluate the behavior of such contaminants during the application of a specific remediation method. The electroremediation method was tested at the laboratory scale using two different soil quantities, three different soil contamination degrees and the same current intensity level.

From results, it was observed that independent of the amount of the soil that was treated, the tendency was a higher treatment efficiency after a longer period of time. Even if it the same general trend was observed concerning the treatment efficiency, some differences were identified, especially when the amount of soil to be treated changed: better results in terms of the remediation degree were achieved by using a higher electric intensity only in case of a smaller quantity of contaminated soil; if the soil quantity to be treated increased, the trend was different, except for the test with the highest contamination degree; and the soil contamination degree had a real influence on the results of the remediation solution: the lower the concentration of the contaminant in the soil, the higher the remediation degree of the applied remediation method (apart from the treatment of a high amount of contaminated soil with a 5% soil contamination degree).

In terms of energy consumption, the results showed that an increase of the treated soil quantity of approximately three times caused a decrease in specific energy consumption of approximately 56%.

Overall, this study suggests that if an organic polluted soil possesses the appropriate properties (like appropriate humidity, pH, conductivity, ORP), using electrochemical treatment for soil remediation may be feasible and successful. The study and its results are useful for improving the electrochemical treatment method for proper management of contaminated sites. It can also serve as support for scaling up the proposed solution to field applications, contributing to the development of remediation strategies.
